# Prevalence of metabolic syndrome in people living with HIV and its multi-organ damage: a prospective cohort study

**DOI:** 10.1186/s12879-025-10735-7

**Published:** 2025-03-12

**Authors:** Jia Tang, Ling Chen, Wei Pan, Lianfeng Lu, Xiaosheng Liu, Leidan Zhang, Liyuan Zheng, Xiaojing Song, Fuping Guo, Wei Lv, Wei Cao, Evelyn Hsieh, Taisheng Li

**Affiliations:** 1 Department of Infectious Diseases, Peking Union Medical College Hospital, Chinese Academy of Medical Sciences & Peking Union Medical College, Beijing, 100730 China; 2 Information Center Department, State Key Laboratory of Complex Severe and Rare Diseases, Peking Union Medical College, Chinese Academy of Medical Sciences, Peking Union Medical College Hospital, Beijing, 100730 China; 3 Department of Infectious Disease, The Second Hospital of Nanjing, Nanjing Medical University, Nanjing, 210003 China; 4 School of Medicine, Tsinghua University, Beijing, China; 5 Section of Rheumatology, Department of Internal Medicine, Allergy and Immunology, Yale School of Medicine, New Haven, CT 06510 USA; 6 State Key Laboratory of Complex Severe and Rare Diseases, Peking Union Medical College Hospital, Chinese Academy of Medical Science and Peking Union Medical College, Beijing, 100730 China; 7 Department of Basic Medical Sciences, School of Medicine, Tsinghua University, Beijing, 100084 China; 8 Tsinghua-Peking Center for Life Sciences, Beijing, 100084 China

**Keywords:** HIV, Metabolic syndrome, Cardiovascular disease, chronic kidney disease, Bone health

## Abstract

**Introduction:**

With the global scale-up of antiretroviral therapy (ART) and improved life expectancy, people living with HIV (PLWH) increasingly face non-infectious comorbidities, and metabolic syndrome (MetS) is one of the most prevalent. MetS is associated with unfavorable health outcomes, including cardiovascular disease, chronic kidney disease, and metabolic dysfunction-associated fatty liver disease. However, data on the prevalence and risk factors of MetS among treatment-naïve PLWH in China are limited. The aim was to investigate the prevalence and risk factors of MetS and to understand its association with multi-organ damage.

**Methods:**

Data on sociodemographic, physical, and clinical characteristics were collected from a completed multicenter, prospective cohort study in China. MetS was diagnosed according to criteria proposed by the China Diabetes Society. Univariate and multivariable logistic regression were applied to identify associated risk factors for MetS. The relationship with organ damage, including kidney, liver, heart, and bone health, were also been assessed.

**Results:**

Among the 449 participants (median age 30 years; 92.9% male), 16.9% met the criteria for MetS. Patients met MetS criteria in our study presented with low HDL-C concentration (49.8%), hypertriglyceridemia (26.1%), hypertension (23.1%), hyperglycemia (15.4%), and abdominal obesity (8.0%). Risk factors significantly associated with MetS included older age (OR 1.08; 95% CI 1.02–1.15) and alcohol consumption (OR 3.63; 95% CI 1.13–11.67). PLWH with MetS exhibited higher level of organ involvement, including reduced kidney function, elevated liver enzymes, and increased risks for cardiovascular events. Among them, 162 participants (36.0%) were classified as being at moderate or high risk using pooled cohort equations (PCEs). It is worth noting that in the MetS group, the dropped bone mineral density (BMD) in the spine decreased more significantly than that of the non-MetS group (*P* = 0.007).

**Conclusion:**

The incidence of MetS in ART-naïve PLWH in China is relatively high. Older age and alcohol consumption are associated with higher risk of MetS. Multiple organ damage may occur accompanied with MetS. Early identification and intervention are critical in managing MetS in PLWH.

**Supplementary Information:**

The online version contains supplementary material available at 10.1186/s12879-025-10735-7.

## Introduction

Given the global scale-up of antiretroviral therapy (ART) and improved life expectancy, people living with HIV (PLWH) are increasingly confronted with non-infectious comorbidities [1, 2]. Indeed, cardiovascular diseases, metabolic complications, cancer, and bone disorders are the most common comorbidities in long-treated PLWH [3]. Among these, metabolic syndrome (MetS) is one of the most frequent [4]. MetS, a complex disease spectrum with various metabolic and cardiovascular risk factors, is mainly characterized by disorders in carbohydrate, protein, and lipid metabolism [5, 6]. Previous studies have shown that various risk factors, such as alcohol consumption, undernutrition, and even family living habits, may contribute to vulnerability to MetS [7]. Nowadays, MetS has become a worldwide epidemic and a major public health concern due to its multiple impacts, including increasing incidence of myocardial infarction, stroke, sudden cardiac death, and tumors [8]. Besides, other diseases such as osteoporosis/osteopenia, chronic kidney disease (CKD), and metabolic dysfunction-associated fatty liver disease (MAFLD) are more likely to develop and progress alongside MetS [8–10].

Many studies have confirmed that the prevalence of MetS is higher in PLWH compared to the general population [11]. The prevalence of MetS among PLWH is estimated at 11–48% globally, and were reported 23.9% (95% CI 19.5–28.7) in Africa in meta-analyses [12, 13]. However, the reported prevalence of MetS varied due to differences in diagnostic criteria for MetS [11, 14] and different underlying population characteristics. Many international organizations and expert groups, such as the World Health Organization (WHO), the European Group for the study of Insulin Resistance (EGIR), the National Cholesterol Education Program Adult Treatment Panel III (NCEP: ATPIII), the American Association of Clinical Endocrinology (AACE), and the International Diabetes Federation (IDF) have attempted to incorporate all the different parameters used to define MetS [15]. The criteria proposed by the China Diabetes Society [16] are widely used in China. However, lack of a unified definition for MetS and the complexity in risk factors have presented a great barrier in studies comparing MetS in PLWH versus the general population. Meanwhile, the incidence and risk factors of MetS are well documented in developed countries, but the data in China are limited [17].

In this study, we estimated the prevalence of MetS in PLWH in a multicenter, prospective cohort in China. The presentation and risk factors of MetS were determined in this population. Evidence on epidemiological, pathogenetic and clinical data on MetS in HIV infection could provide new lights of prevention strategies and therapeutic options in the future.

## Methods

### Study design and participants

Data were drawn from the China AIDS Clinical Trial (CACT)1807 study (NCT03598556, 2020-07-07). This was a prospective, multicenter cohort study that enrolled newly diagnosed PLWH before ART initiation at 6 clinical sites in China between January 2017 and December 2020. The CACT 1807 study was originally designed to assess the efficacy and safety of vitamin D supplementation in PLWH initiating the regimen of lamivudine (3TC)-tenofovir disoproxil fumarate (TDF)-efavirenz (EFV).

**Fig. 1:**
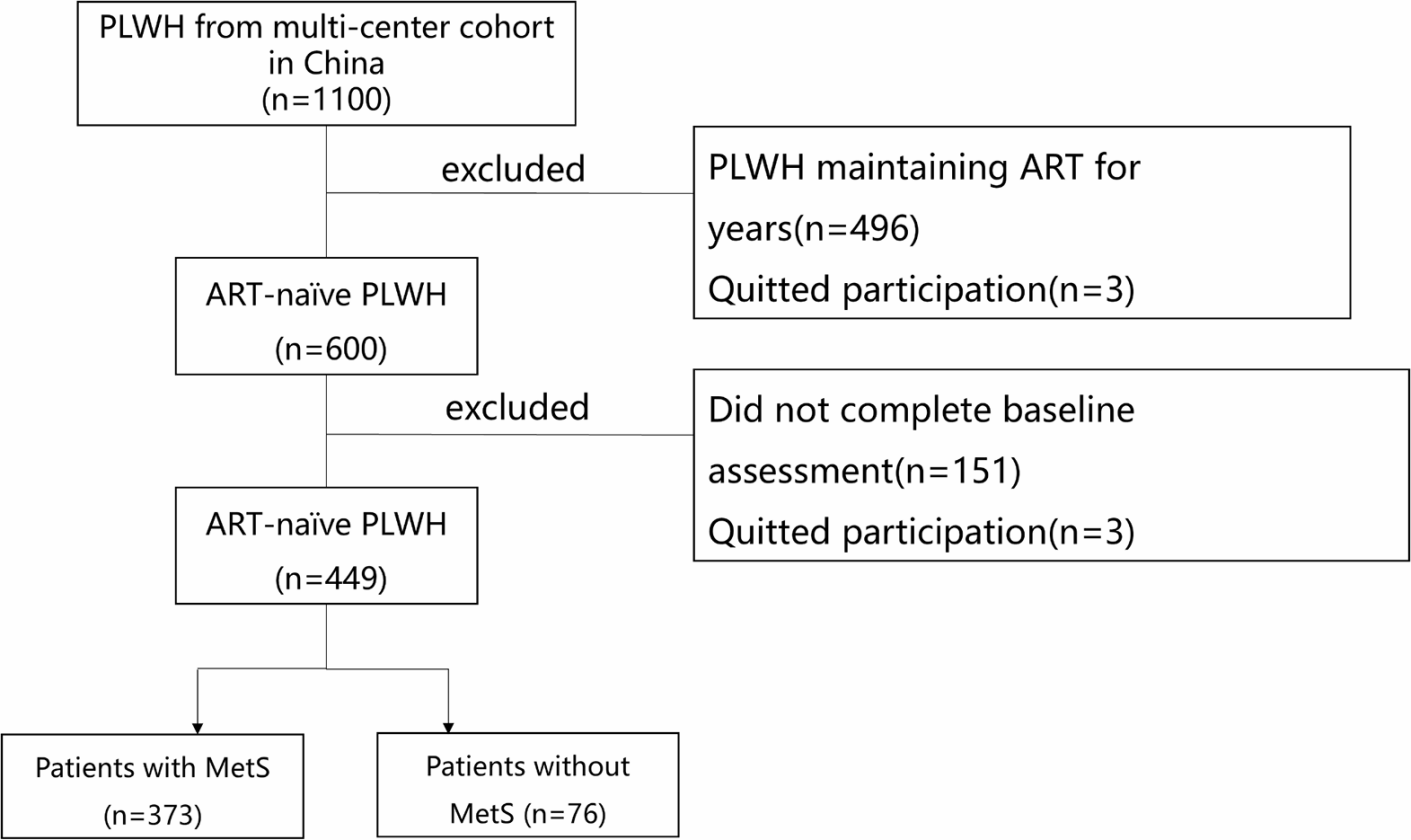
Flow chart of included participants

For the current study, we collected the baseline sociodemographic and clinical data at study entry. Participants who had bone mineral density (BMD) evaluations at baseline with at least one-year follow-up were included. The study received approval from an independent ethics committee and the institutional review board of Peking Union Medical College Hospital (PUMCH) (No.1-23PJ189). All participants provided written informed consent. During the enrollment phase of the CACT study, we consecutively screened ART-naïve PLWH. Figure 1 outlines the screening process, resulting in 449 participants included in the final analysis.

### Data collection and variable definitions

Baseline sociodemographic and clinical data were obtained from 449 consenting participants and recorded in a password-protected spreadsheet accessible only to the research team. All personal identifiers, including names and identification numbers, were removed to maintain confidentiality. Clinical characteristics were extracted including gender, age, body mass index (BMI), level of education, occupation, religion, smoking status, alcohol consumption, physical activity, blood pressure, vitamin D supplementation, HIV transmission route, viral load (log10 copies/ml), and baseline CD4 + T cell count (cells/mm³). Waist circumference (WC) measurements were used to assess abdominal obesity, measured in a standing position at the level of the navel, using a non-elastic, plastic tape measure. Measurements were taken three times, and the average value was used for analysis to minimize error. All measurements were performed by trained research staff following standard protocols to ensure consistency and accuracy. Liver enzymes, including alanine aminotransferase (ALT), aspartate aminotransferase (AST), alkaline phosphatase (ALP), gamma-glutamyl transpeptidase (GGT). BMDs of the lumbar spine (LS), femoral neck (FN), and total hip (TH) were obtained for each participant using dual-energy X-ray absorptiometry (DXA) scan equipment (GE Lunar Prodigy Advanced scanner, GE Healthcare, Madison, WI) and the same GE Lunar software (enCORE version 10.50.086).

### Definition

#### Definition of MetS

Metabolic Syndrome (MetS) was defined according to the China Diabetes Society guidelines [16]. Individuals meeting at least three of the following criteria were classified as having MetS: (1) Abdominal obesity: waist circumference ≥ 102 cm in men and ≥ 88 cm in women; (2) Hyperglycemia: fasting blood glucose ≥ 6.1 mmol/L or a diagnosis of diabetes; (3) Hypertension (systolic blood pressure ≥ 130 mmHg and/or diastolic blood pressure ≥ 85 mmHg) or a history of hypertension; (4) Hypertriglyceridemia: triglycerides ≥ 1.70 mmol/L; (5) Low HDL-C: < 1.04 mmol/L.

#### CVD risk

Pooled cohort equations (PCEs) were used to compare CVD risk in the next 10 years in people living with HIV with or without MetS [18]. The 10-year atherosclerotic CVD risk scores were categorized as low risk (< 5%), moderate risk (5–9%), and high risk (≥ 10%).

### Statistical analysis

All statistical analyses were performed using R 4.3.0 (https://www.r-project.org/). Continuous variables are expressed as the median and interquartile range (IQR). Categorical variables are presented as the number and percentage (%). The chi-square test or Fisher’s exact test was used for comparisons between categorical variables, while the t-test or Mann-Whitney U test was used for continuous variables, as appropriate. Associations between MetS and its components (i.e., hypertension, dyslipidemia, and hyperglycemia) with potential risk factors and organ damage were analyzed using logistic regression models, reporting odds ratios (ORs) and 95% confidence intervals (CIs). Sensitivity analyses were performed to assess the robustness of the results, by excluding individuals with hepatitis B and C co-infection (defined as HBsAg or hepatitis C virus antibody positive), alcohol use and participants with baseline CD4 + T cell counts under 200 cells/mm³ to reduce potential selection bias. All tests were two-sided, with statistical significance set at *P* < 0.05.

## Results

### Demographic characteristics

A total of 449 PLWH were enrolled in the study, with the majority being male (92.9%, 417/449) (Fig. 1). The median age (IQR) was 30 (26–39) years. Over half participants had a high level of education or higher (62.6%, 281/449), were of Han ethnicity (88.0%, 281/449), were homosexual (68.4%, 307/449), and were employed (92.7%, 416/449). The median CD4 + T cell count (IQR) at enrollment was 370 (252–508) cells/mm³. All participants were intended to be treated with an ART regimen of 3TC-TDF-EFV. A total of 76 patients (16.9%) met the criteria for MetS. The composition of MetS, listed by prevalence, were low HDL-C concentration (49.8%), hypertriglyceridemia (26.1%), hypertension (23.1%), hyperglycemia (15.4%), and abdominal obesity (8.0%) (Table 1).

**Table 1:** Baseline demographics and clinical characteristics by MetS group

Variables	Total (*n* = 449)	Non-MetS (*n* = 373)	MetS (*n* = 76)	*P*
Age(years)	30.00 (26.00, 39.00)	29.00 (25.00, 36.00)	36.00 (28.00, 46.50)	**< 0.001**
Gender, male, n(%)	417 (92.87)	348 (93.30)	69 (90.79)	0.439
BMI (kg/m^2^)	21.91 (20.05, 24.22)	21.43 (19.72, 23.18)	25.68 (23.32, 27.14)	**< 0.001**
Waist Circumference (cm)	85.00 (79.00, 92.00)	83.00 (78.00, 89.00)	94.00 (90.00, 100.00)	**< 0.001**
Hip Circumference (cm)	95.00 (91.00, 100.00)	94.00 (90.50, 99.00)	102.00 (98.00, 106.00)	**< 0.001**
HGB(g/L)	149.00 (138.00, 159.00)	149.00 (138.75, 159.00)	148.00 (137.75, 157.25)	0.709
WBC(*10^9^/L)	5.26 (4.42, 6.42)	5.25 (4.44, 6.42)	5.42 (4.36, 6.43)	0.916
Lymphocyte (%)	31.85 (23.37, 39.52)	31.50 (22.37, 39.20)	33.05 (27.17, 41.68)	0.055
PLT(*10^12^/L)	216.00 (179.00, 247.25)	215.00 (180.00, 245.25)	227.50 (175.25, 253.50)	0.609
ALT(U/L)	21.00 (14.00, 32.00)	20.00 (13.70, 30.00)	28.70 (17.30, 41.10)	**< 0.001**
AST(U/L)	21.50 (18.00, 27.00)	21.00 (17.80, 25.30)	25.00 (18.93, 32.00)	**0.004**
GGT(U/L)	22.00 (14.80, 34.00)	20.00 (14.00, 31.00)	30.15 (20.95, 48.70)	**< 0.001**
ALP(U/L)	73.00 (63.00, 85.00)	73.75 (63.30, 85.22)	71.05 (59.00, 83.12)	0.349
Cr (umol/L)	72.00 (64.70, 79.00)	72.00 (64.40, 79.00)	71.70 (65.85, 79.25)	0.568
eGFR(mL/min/1.73m^2^)	110.25 (97.80, 124.28)	111.52 (98.69, 125.33)	107.17 (90.59, 118.64)	**0.028**
eGFR < 90mL/min/1.73m^2^, n(%)	71 (15.81)	54 (14.48)	17 (22.37)	0.086
FBG (mmol/L)	5.33 (4.99, 5.75)	5.27 (4.97, 5.61)	6.08 (5.43, 6.62)	**< 0.001**
TG (mmol/L)	1.16 (0.82, 1.73)	1.06 (0.75, 1.50)	2.16 (1.76, 2.95)	**< 0.001**
CHO (mmol/L)	4.02 (3.53, 4.53)	4.01 (3.54, 4.52)	4.13 (3.51, 4.56)	0.411
LDL (mmol/L)	2.35 (1.97, 2.79)	2.36 (1.99, 2.80)	2.29 (1.80, 2.77)	0.470
HDL (mmol/L)	1.00 (0.85, 1.20)	1.06 (0.88, 1.25)	0.83 (0.73, 0.91)	**< 0.001**
Baseline viral load (log10copies/ml)	4.58 (4.18, 5.05)	4.57 (4.19, 5.01)	4.66 (4.12, 5.29)	0.333
Baseline CD4 + T cell count (cell/mm^3^)				
Median (IQR)	370.00 (252.00, 508.00)	363.00 (253.00, 505.00)	396.74 (243.50, 518.25)	0.635
<200	65 (14.48)	53 (14.21)	12 (15.79)	0.850
200–500	267 (59.47)	224 (60.05)	43 (56.58)	
>500	117 (26.06)	96 (25.74)	21 (27.63)	
Ethnicity, n (%)				0.269
Han	395 (87.97)	331 (88.74)	64 (84.21)	
Others	54 (12.03)	42 (11.26)	12 (15.79)	
Highest level of education, n (%)				0.088
Under high school	168 (37.42)	133 (35.66)	35 (46.05)	
College/university	281 (62.58)	240 (64.34)	41 (53.95)	
Employment, n (%)				0.495
Yes	416 (92.65)	347 (93.03)	69 (90.79)	
No	33 (7.35)	26 (6.97)	7 (9.21)	
Route of Transmission, n (%)				**0.044**
Homosexual/Heterosexual	307 (68.37)	264 (70.78)	43 (56.58)	
Blood transfusion	89 (19.82)	67 (17.96)	22 (28.95)	
Unknown	53 (11.80)	42 (11.26)	11 (14.47)	
Urine albumin, n (%)				0.074
No	384 (85.52)	314 (84.18)	70 (92.11)	
Yes	65 (14.48)	59 (15.82)	6 (7.89)	
Smoking, n(%)				0.951
Never	254 (57.86)	211 (57.97)	43 (57.33)	
Past smoked	62 (14.12)	52 (14.29)	10 (13.33)	
Currently smoking	123 (28.02)	101 (27.75)	22 (29.33)	
Alcohol consumption, n(%)				**0.029**
Yes	46 (33.58)	42 (37.84)	4 (15.38)	
No	91 (66.42)	69 (62.16)	22 (84.62)	
Vitamin D supplementation	224 (49.89)	186 (49.87)	38 (50.00)	0.983
Physical exercise				0.091
Yes	147 (33.72)	128 (35.46)	19 (25.33)	
Not active	289 (66.28)	233 (64.54)	56 (74.67)	

Abbreviations: IQR, interquartile range; ART, antiretroviral therapy; BMI, body mass index; HGB: hemoglobin; WBC: white blood cell; PLT: platelets; ALT: alanine aminotransferase; AST: aspartate aminotransferase; ALP: alkaline phosphatase; GGT: gamma-glutamyl transpeptidase; Cr: creatinine; eGFR, estimated glomerular filtration rate; FBG: fasting blood glucose; TG, triglycerides; CHO: total cholesterol; LDL: low-density lipoprotein cholesterol; HDL: high-density lipoprotein cholesterol

### Factors associated with MetS

**Fig. 2:**
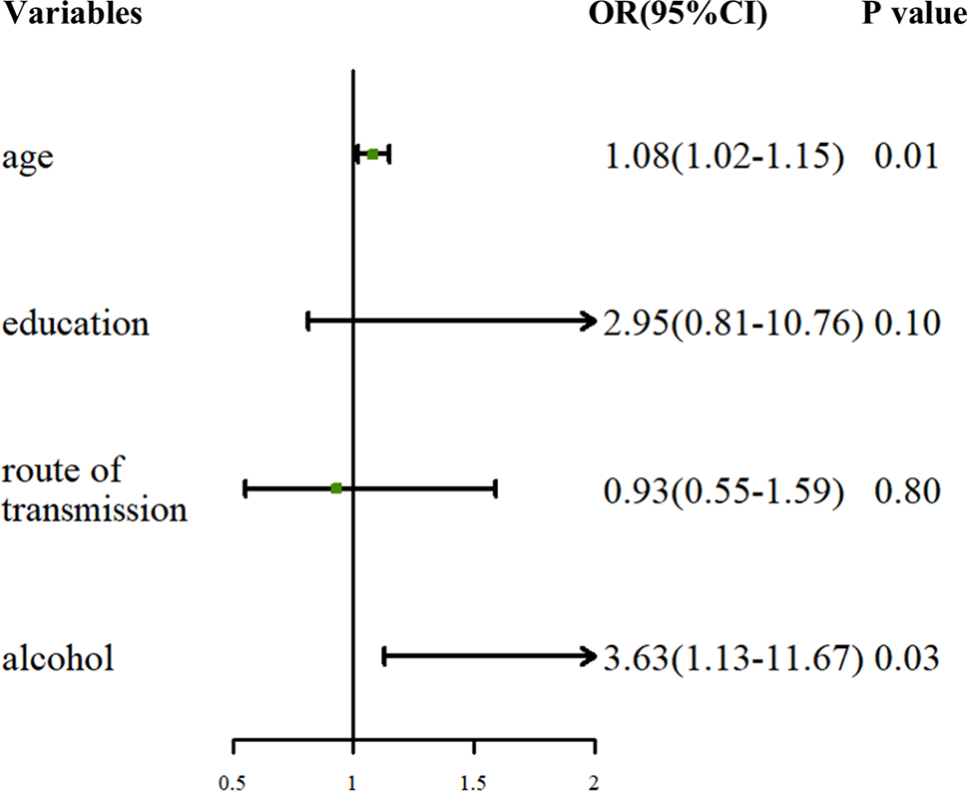
Forest plot of factors associated with metabolic syndrome (multivariable analysis)

Table 1 presents the results of the univariate analysis of factors associated with MetS, including sociodemographic, behavioral, physical, and clinical characteristics. Factors associated with a higher prevalence of MetS included age, routes of transmission, and alcohol consumption. Additionally, compared with patients without MetS, those with MetS had significantly higher levels of abnormal liver enzymes (ALT, AST, GGT, and ALP) and renal dysfunction. In the adjusted multivariable logistic regression analysis, older age (OR = 1.08; 95% CI 1.02–1.15) and alcohol consumption (OR = 3.63; 95% CI 1.13–11.67) were significantly associated with MetS (Fig. 2).

### Association between MetS and target organ damage

We further assessed the organ impact of MetS, including the kidney, liver, heart, and bone. The proportion of participants with an estimated glomerular filtration rate (eGFR) < 90 mL/min/1.73 m² was higher in the MetS group than in the non-MetS group (22.4% vs. 14.5%), and the median eGFR (IQR) was lower in the MetS group than in the non-MetS group (107.2 [90.6-118.6] vs. 111.5 [98.7-125.3], *P* < 0.05). Univariate analysis revealed a significant association between positive urine albumin and MetS (*P* = 0.008). Liver enzymes, including ALT, AST, and GGT, were notably elevated in the MetS group. Specifically, the median ALT (IQR) was significantly higher (28.7 [17.3–41.1] vs. 20.0 [13.7–30.0], *P* < 0.001), as was the median AST (IQR) (25.0 [18.9–32.0] vs. 21.0 [17.8–25.3], *P* = 0.004) and the median GGT (IQR) (30.2 [21.0-48.7] vs. 20.0 [14.0–31.0], *P* < 0.001). However, there was no significant difference in the median ALP (IQR) between the groups (71.1 [59.0-83.1] vs. 73.8 [63.3–85.2], *P* > 0.05). The logistic regression analysis indicated a positive association between MetS and elevated ALT levels (*P* < 0.05). Regression models were applied to assess the outcomes of (1) ALT levels exceeding the upper limit of normal (ULN), defined as 50 U/L for men and 40 U/L for women, and (2) the degree of ALT elevation above ULN at the most recent visit (Table 2). The two aspects of results both showed a positive link between abnormal ALT and MetS (*P* < 0.05).

**Table 2:** Comparison of target organ function between the metabolic syndrome (MetS) group and non-MetS group

Variables	β	S.E	Z	*P*	OR (95%CI)
Urine albumin					
Negative					1.00 (Reference)
Positive	-1.10	0.42	-2.66	**0.008**	0.33 (0.15 ~ 0.75)
eGFR < 90 mL/min/1.73m^2^					
No					1.00 (Reference)
Yes	0.31	0.31	1.01	0.313	1.36 (0.75 ~ 2.49)
eGFR(mL/min/1.73m^2^)	-0.01	0.01	-1.58	0.114	0.99 (0.98 ~ 1.00)
ALT(U/L)					
Normal					1.00 (Reference)
Elevated	0.66	0.25	2.60	**0.009**	1.94 (1.18 ~ 3.19)
ALT(ULN)					
Normal					1.00 (Reference)
<2ULN	0.41	0.28	1.44	0.151	1.50 (0.86 ~ 2.62)
<3ULN	1.37	0.42	3.25	**0.001**	3.93 (1.72 ~ 8.98)
>3ULN	1.57	0.78	2.01	**0.045**	4.82 (1.04 ~ 22.42)

Abbreviations: OR: Odds Ratio, CI: Confidence Interval; eGFR, estimated glomerular filtration rate; ALT: alanine aminotransferase; ULN: upper limit of normal

Note: the ULN of ALT was > 40U/L for women or > 50U/L for men

**Fig. 3:**
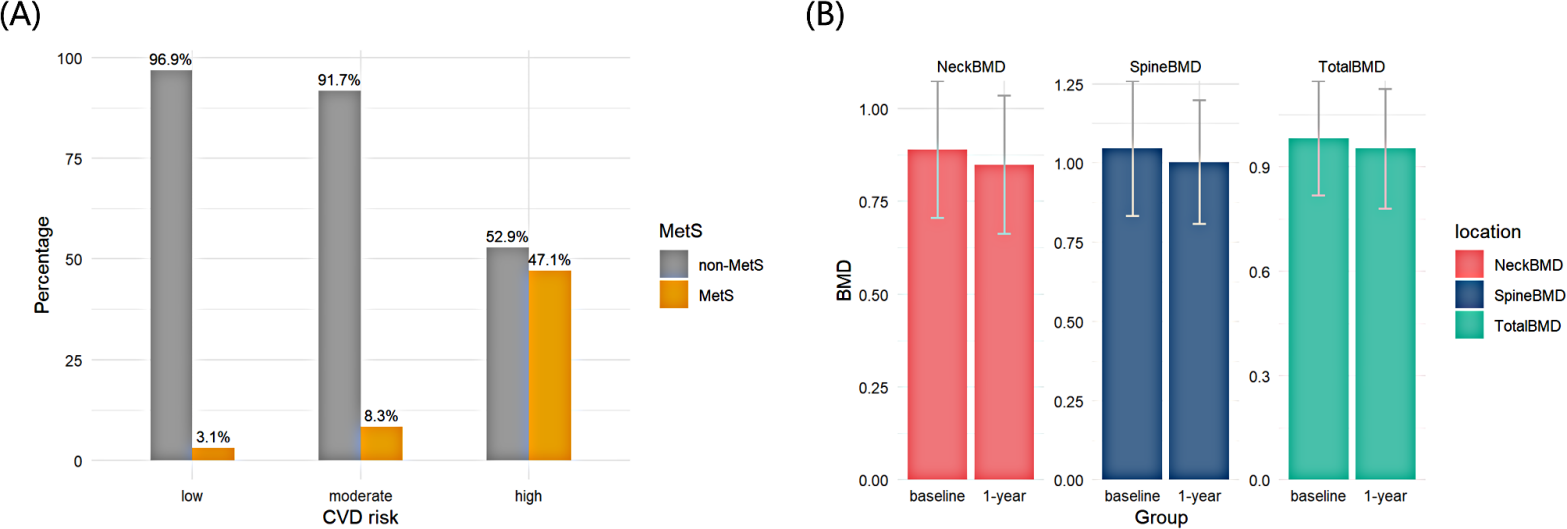
(**A**) Comparison of cardiovascular disease risk classification between the groups with (MetS) and without (non-MetS) metabolic syndrome in the next 10 years. (**B**) Comparison of BMD in different detection locations in the baseline and 1 year after

When assessing the 10-year risk of developing cardiovascular disease (CVD) events using pooled cohort equations (PCEs), 162 participants (36.0%) were classified as being at moderate or high risk. The proportion of patients at very high risk for CVD events was significantly greater in the MetS group compared to the non-MetS group, with 65/76 (85.53%) versus 73/373 (19.57%), respectively (Fig. 3A). Overall, the MetS group had a significantly higher proportion of patients at high or very high risk for CVD events in the next 10 years compared to the non-MetS group (*P* < 0.001).

Among the 201 patients who completed two bone mineral density (BMD) assessments with the 3TC-TDF-EFV regimen, there was consistent bone loss in both femur neck and spine BMD measurements (*P* < 0.05 for both) (Fig. 3B). Between baseline and one-year follow-up, spine BMD decreased by 0.08 g/m^2^ in the MetS group and by 0.01 g/m^2^ in the non-MetS group (*P* = 0.007). However, there was no significant difference in femur neck BMD and total BMD(Table 3).

**Table 3:** Decreases in BMD by site between the metabolic syndrome (MetS) group and non-MetS group

Variables	Total (*n* = 201)	Non-MetS(*n* = 161)	MetS (*n* = 40)	*P*
Neck BMD	0.03 (-0.07, 0.12)	0.03 (-0.08, 0.12)	0.03 (-0.03, 0.13)	0.525
Total BMD	0.04 (-0.07, 0.11)	0.03 (-0.09, 0.11)	0.05 (-0.04, 0.16)	0.281
spine BMD	0.03 (-0.08, 0.12)	0.01 (-0.10, 0.11)	0.08 (0.00, 0.18)	**0.007**
Neck T-Score	0.20 (-0.50, 1.00)	0.20 (-0.50, 0.90)	0.30 (-0.23, 1.15)	0.396
Neck Z-Score	0.20 (-0.60, 1.10)	0.20 (-0.60, 0.90)	0.35 (-0.10, 1.23)	0.179
Total T-Score	0.20 (-0.50, 0.80)	0.10 (-0.60, 0.70)	0.40 (0.00, 1.22)	0.089
Total Z-Score	0.20 (-0.50, 0.90)	0.20 (-0.60, 0.80)	0.40 (-0.10, 1.22)	0.085
spine T-Score	0.20 (-0.80, 1.10)	0.10 (-0.90, 1.00)	0.70 (0.08, 1.63)	**0.006**
spine Z-Score	0.20 (-0.80, 1.10)	0.10 (-0.90, 1.00)	0.70 (0.10, 1.60)	**0.004**

Note: presented with Median and interquartile

### Sensitivity analysis

Considering the potential confounding factors between ALT and MetS, a sensitivity analysis was conducted by excluding participants with HBV/HCV coinfections and alcohol use (*n* = 67) and those with nadir CD4 + T cell counts < 200 cells/mm³ (*n* = 103). After balancing the confounders, The associations remained consistent, underscoring the stability of the findings (eTables 1 and 2 in the Supplementary).

## Discussion

In our multicenter study, the overall prevalence of MetS among Chinese PLWH before receiving antiretroviral therapy (ART) was 16.9%, which was higher compared to reported data in other parts of the world (15.2% in Kenya [19] and 13.9% in Africa [20]). Globally, the prevalence of MetS among PLWH ranges from 24.5–35.1% [12, 21]. The prevalence of MetS among the general Chinese population was found to be 21.25–38.63% according to different definitions [22]. Variations in MetS prevalence among studies could be attributed to different definitions of MetS. Furthermore, considering the younger median age (30 years), and limited period of follow-up (only one year) in our cohort, the prevalence of MetS may be underestimated. Meanwhile, our study reported a prevalence rate of 23.6% (106 out of 449) according to the NCEP: ATP III criteria. In contrast, a smaller cross-sectional study reported a prevalence of 5.92% based on the same criteria [23].The prevalence may further increase when incorporating metabolic impacts from various long-term ART regimens. For example, Liu et al. [17] reported a prevalence of 33.9% in PLWH on long-term ART in China.

In our study, traditional MetS risk factors were assessed using adjusted multivariable logistic regression analysis. Older age (OR = 1.08; 95% CI 1.02–1.15) and alcohol consumption (OR = 3.63; 95% CI 1.13–11.67) were significantly associated with MetS, which aligns with previous studies [17, 20, 24]. In addition, previous studies also pointed out that male, significant weight gain since the age of 20, current smoking, slow walking speed, and fast eating speed were independently related to multiple MetS components. Besides, both traditional factors and specific single nucleotide polymorphisms (SNPs) play a significant role in predicting MS in a recent study using machine learning. For men, alcohol consumption and the genetic variant rs11216126 were evident, whereas for women, dietary intake and the genetic variant rs780094 were more significant [25].

Consistent with previous studies [26], we did not find HIV-specific factors potentially associated with MetS, including baseline CD4 + T cell counts or viral load. The pathophysiological mechanisms linking MetS with ART-naïve PLWH are not fully elucidated. HIV-specific factors such as immune activation and chronic inflammation, interacting with traditional risk factors (e.g., smoking, poor diet, sedentary lifestyle), drive MetS in aging PLWH simultaneously in separate or synergic ways [11, 28]. Additionally, chronic inflammation and oxidative stress which are common in PLWH may promote insulin resistance and endothelial dysfunction, further contributing to MetS development [28]. However, our cohort was ART-naïve, avoiding therapeutic confounding factors such as ART regimens, duration of ART, and immune reconstitution status.

Meanwhile, organ damage could be influenced and regulated by these factors as well. MetS is associated with an increased risk of renal injury, diabetes, fatty liver disease, cardiovascular events, and mortality [27–29]. Although our study did not find a significant association between MetS and chronic kidney disease (CKD), the presence of proteinuria suggested early kidney damage, according to previous studies [17]. This may result from the imbalance of glucose and lipid metabolism regulated by the kidney, which involves in MetS pathogenesis through hemodynamic changes, sympathetic nerve excitation, increased reactive oxygen species (ROS) production, renin-angiotensin-aldosterone system (RAAS) activation, and adipokine abnormalities due to insulin resistance (IR), obesity, hypertension, and hyperlipidemia [30, 31]. Furthermore, persistent liver inflammation (indicative of potential liver disease) was observed in one-third of participants without viral hepatitis, drug-induced liver injury, or heavy alcohol use. These findings were constant in sensitive analysis and consistent with previous research [29, 32]. Moreover, we also assessed the 10-year cardiovascular disease (CVD) risk using Pooled Cohort Equations (PCEs), finding a marginally increased HIV-associated CVD risk in the MetS group, consistent with previous research [17]. Indeed, the calculations for CVD risk were complex and varied, and were not conducted as frequently as necessary. Further cohort studies are needed to investigate in this issue.

Antiretroviral therapy (ART) drugs might contribute to accelerated bone loss. Previous studies have shown a strong and consistent association between TDF exposure and bone deficits [33, 34]. MetS can also contribute to bone loss in the general population [35]. Although studies did not provide direct evidence for the mechanistic role of MetS in bone loss, several hypotheses are worth considering. Obesity and insulin resistance generally have a negative impact on BMD, considering their association with systemic inflammation, increased inflammatory cytokines, and regulatory hormones for bone metabolism [36, 37]. A study revealed a positive correlation between MetS and BMD at the pelvis (β: 0.046 [95% CI 0.02–0.07]), thoracic spine (β: 0.047 [95% CI 0.02–0.07]), and lumbar spine (β: 0.040 [95% CI 0.02–0.06]) [35]. However, BMD can be influenced by various factors, including diet, physical activity and vitamin D supplementation [38]. To reduce confounding factors, we compared the baseline characteristics, which yielded comparable results. However, interpreting these findings is complicated by the inherent limitations of observational studies and the complex interactions involved, which make them susceptible to confounding factors and reverse causation. To address these challenges, statistical methods such as inverse probability weighting (IPW) and Mendelian randomization (MR) analysis are employed, particularly in studies with large sample sizes [39].

Our study had several limitations worthy of discussion. Firstly, the definition of MetS and its key components is based on models specific to China with limited evidence in foreign populations, which may exhibit significant phenotypic and genotypic differences. Secondly, the sample size was relatively small, and we did not include a control population of people without HIV from the same region for comparison. Thirdly, the research period was relatively short, preventing us from including the gold standard for CVD events and lacking imaging checks, such as carotid artery and cardiac ultrasonography. Additionally, osteoporosis is a long-term process, and the decrease in BMD within the first year can be subtle. Further research is warranted. Lastly, abnormal liver enzyme levels could be influenced by several confounding factors, such as side effects of drugs and co-infections. In this regard, we presented a sensitivity analysis, which did not change the observed link.

## Conclusions

In conclusion, this study highlights the significant incidence of metabolic syndrome in ART-naïve PLWH in China, and shows that older age and alcohol consumption have significant associations with metabolic syndrome. The presence of MetS correlates with increased liver enzyme abnormalities and renal dysfunction, bone loss, and a higher risk of cardiovascular events. These findings emphasize the urgency of integrating MetS screening and management into HIV care protocols to mitigate long-term health risks. Public health resources should focus on early intervention strategies and continuous monitoring to address MetS in PLWH, ultimately improving overall health outcomes and life expectancy in this vulnerable population. Further research is needed to explore the underlying mechanisms and long-term impacts of MetS in PLWH, particularly in the context of ART initiation and progression.

## Electronic supplementary material

Below is the link to the electronic supplementary material.

Supplementary Material 1

## Data Availability

All data generated or analyzed during this study are included in this published article.

## Abbreviations

PLWH: People living with HIV
MetS: Metabolic syndrome
ART: Antiretroviral therapy
BMD: Bone mineral density
CVD: Cardiovascular disease
PCEs: Pooled cohort equations
3TC: Lamivudine
TDF: Tenofovir disoproxil fumarate
EFV: Efavirenz
