# Supplementary material for: Prevalence of metabolic syndrome in people living with HIV and its multi-organ damage: a prospective cohort study
Source: BMC Infect Dis. 2025 Mar 12;25:351. doi: 10.1186/s12879-025-10735-7 (PMC11905433; doi:10.1186/s12879-025-10735-7)
Supplement: Supplementary file 1 — Supplementary Material 1 [file 12879_2025_10735_MOESM1_ESM.docx]

eTable1. The association between ALT and MetS excluding HBV/HCV/heavy alcohol

| Variables | Model 1 | | Model 2 | | Model 3 | |
| --- | --- | --- | --- | --- | --- | --- |
|  | OR (95%CI) | *P* | OR (95%CI) | *P* | OR (95%CI) | *P* |
| ALT |  |  |  |  |  |  |
| Normal | 1.00(Reference) |  | 1.00(Reference) |  | 1.00(Reference) |  |
| Elevation | 2.09(1.14-3.81) | **0.016** | 2.20(1.19-4.05) | **0.012** | 2.10(1.14-3.89) | **0.018** |

Abbreviations: OR: Odds Ratio, CI: Confidence Interval;

Model 1: adjusted for age, gender;

Model 2: adjusted for model 1 + education status, route of transmission

Model 3: adjusted for model 2 + viral load, CD4+ T cell count

eTable2. The association between ALT and MetS excluding nadir CD4 count <200

| Variables | Model 1 | | Model 2 | | Model 3 | |
| --- | --- | --- | --- | --- | --- | --- |
|  | OR (95%CI) | *P* | OR (95%CI) | *P* | OR (95%CI) | *P* |
| ALT |  |  |  |  |  |  |
| Normal | 1.00(Reference) |  | 1.00(Reference) |  | 1.00(Reference) |  |
| Elevation | 1.98(1.09-3.58) | **0.024** | 1.93(1.06-3.54) | **0.031** | 1.97(1.14-3.70) | **0.035** |

Abbreviations: OR: Odds Ratio, CI: Confidence Interval;

Model 1: adjusted for age, gender;

Model 2: adjusted for model 1 + education status, route of transmission

Model 3: adjusted for model 2 + viral load, CD4+ T cell count
